# Supplementary material for: A qualitative exploration of video-based motor action observation perceptions in patients with chronic low back pain and asymptomatic participants: An interpretative phenomenological analysis
Source: PLoS One. 2026 Feb 6;21(2):e0326638. doi: 10.1371/journal.pone.0326638 (PMC12880644; doi:10.1371/journal.pone.0326638)
Supplement: S1 File — The final version of the guide is presented, including the complete information on all items and probing questions. (DOCX) [file pone.0326638.s001.docx]

**Semi-Structured Interview Guide**

*Study title: A Qualitative Exploration of Video-Based Motor Action Observation Perceptions in Patients with Chronic Low Back Pain and Asymptomatic Participants
Intended use: Supplementary File (Interview Guide)*

# 1) Purpose and Scope

This guide elicits lived experiences and meaning-making related to watching, and (if applicable) attempting, exercises demonstrated in motor-action videos encountered in participants’ usual environments. It explores: first impressions; attentional focus; comprehension; embodied/feasibility aspects; cognitions and agency; motivation and perceived effects; social/contextual factors; modality preferences; and user-centered recommendations. The interview follows an Interpretative Phenomenological Analysis (IPA) orientation (idiographic focus; event-centered narration; reflexivity). No in-session video stimulus is used.

# 2) Target Participants

Adults with chronic non-specific low back pain (CLBP) and asymptomatic adults (18–65 years) who have watched exercise/motor-action videos on social-media/online platforms at least three times in the past two months. Use neutral, non-pathologizing language in both groups; add group-specific probes only when relevant.

# 3) Setting and Materials

• Setting: Quiet private room (in-person) or secure videoconference (remote).

• Duration: ~45–60 minutes.

• Materials: Audio recorder; consent form; brief demographic form; list of platforms used (e.g., YouTube/Instagram); interviewer field-notes template.

# 4) Ethical and Procedural Notes

• Confirm written informed consent before starting.

• Remind participants they may pause/skip any question or stop at any time without consequence.

• Use participant codes only (e.g., PP# for pain group; AP# for asymptomatic).

• Audio-record only with explicit permission; state where files are stored and for how long.

• Data controller/contact: [Institution/PI email]. Legal basis: research with consent/[public interest] per CEI approval [code]. Retention: [X years].

• Rights: access/rectification/erasure where applicable; withdrawal procedure.

• Distress/pain protocol: offer breaks; stop recording if requested; provide brief grounding; signpost support pathways; record an incident note (no clinical treatment is provided).

• Researcher keeps reflexive notes after each interview (assumptions, decisions, notable reactions).

# 5) Interviewer Opening Script (read verbatim)

“Thank you for taking part. I’m interested in your experience of watching exercise or motor-action videos in your everyday life and, if you tried any movements, how that was for you. There are no right or wrong answers — I’m interested in your perspective. Your responses are confidential and will be de-identified in any reports. You can skip any question or stop at any time. With your permission, I’ll audio-record so I don’t miss details. Is that okay?”
(Start recording once the participant agrees.)

# 6) Warm-Up / Context

Viewing context: “Which platforms do you usually use to watch exercise videos (e.g., YouTube, Instagram)? About how often have you watched such videos in the last two months?”

Probe: “Do you typically watch on a phone, tablet, or computer? Do you use captions, playback speed, or playlists?”

# 6bis) Lived Context (Before–During–After)

“Could you walk me through a recent time you watched one of these videos — what happened before, during, and after?”

Probes: where you were; who (if anyone) was around; time of day; what you were hoping for; practical constraints (space/equipment/time).

# 7) Core Domains and Questions (use probes as needed; avoid leading)

## A. First Impressions

1. “What was the very first thing you thought or felt when you watched the exercise videos for the first time?”

Probes: surprise, curiosity, confidence, concern (if any).

2. “How would you describe your overall experience of watching these exercise or motor-action videos?”

Probes: what made it engaging or difficult (if anything).

## B. Attention and Salient Video Features

3. “When you watch an exercise video, what specific elements catch your attention?”

Probes: instructions; pacing/tempo; breathing; camera angle/point-of-view; visual framing; on-screen text/captions; cues about safety/progression; step-by-step structure.

4. “Do you prefer a first-person (egocentric) view or a side/front (allocentric) view? Why?”

Probes: which view makes the movement clearer or feel safer?

5. “How did pacing/tempo influence your understanding or confidence?”

Probes: too fast/too slow; pausing/rewinding; countdowns.

6. “How did the way the person is presented (instruction style, clarity of technique, perceived ability level) shape what you thought you could do, if at all?”

## C. Comprehension and Following Along

7. “Did you find any movements particularly difficult to understand or follow? Please tell me about them.”

Probes: what made them hard—pace, camera angle, lack of cues, too much information?

8. “When videos broke movements into steps, what worked or didn’t? Did you notice any error-avoidance cues that helped?”

Probes: one clear goal; 2–3 steps; one mistake to avoid.

## C1. Embodied Experience

9. “As you watched or tried the movements, what did you notice in your body?”

Probes: breath; muscle tension/relaxation; urge to move/stop; comfort/discomfort or pain (if any); energy; confidence.

## D. Trying to Replicate — Emotions and Feasibility

10. “How did you feel when you tried to replicate movements you saw in the videos?”

Probes: confident, hesitant, worried about pain, encouraged?

11. “Were there moments when the exercises felt hard to do (if at all)? What seemed to make it feel that way?”

Probes: specific positions (e.g., flexion, load), speed, balance, getting on/off the floor; clarity of progression.

12. “Did anything in a video help you reframe a movement from ‘risky’ to ‘doable’?”

Probes: language, tone, first ‘easy win,’ reassurance about flare-ups.

13. “If you felt a flare or worry coming on, what did you do in the moment?”

Probes: stop/modify; change range/tempo; switch variant.

## E. Cognitions During Imitation (Self-Talk and Rules)

14. “What kinds of thoughts went through your mind while trying to imitate the movements?”

Probes: safety rules you tell yourself; ‘do’s and don’ts’; anticipating pain; planning modifications.

### E2. Self-Efficacy Shift

15. “Compared with before watching, how did your confidence to try the movement change, and why?”

Probes: specific video features that boosted/reduced confidence.

### E3. Agency & Identity

16. “In what ways, if any, did this experience affect how you see yourself — for example, as someone who can/can’t do these movements?”

## F. Motivation and Perceived Impact

17. “From your experience, how did these videos relate to your motivation to continue exercising (if at all)?”

Probes: what aspects increased or decreased motivation (e.g., credibility, relevance, quick wins).

18. “Have you noticed any changes (if any) in your physical condition or pain level after following exercises learned from the videos?”

Probes: short-term relief, confidence, function; no change; flare-ups — what seemed to influence that?

### F3. Follow-Through & Adherence

19. “Across days/weeks, what helped you keep using the videos, and what got in the way?”

Probes: time, symptoms, reminders, boredom, progressions.

20. “Did you notice any functional changes (e.g., daily tasks feel easier) even if pain didn’t change?”

Probes: standing up, lifting, walking, sleep.

## G. Modality Preference (Videos vs. Other Formats)

21. “Compared with other ways of learning exercises — like live sessions with a therapist — what do you prefer, and why?”

Probes: access, flexibility, feedback, safety, personalization, privacy.

## H. Practical Advice (User-Centered Recommendations)

22. “Based on your experience, what recommendations would you give someone like you about using videos to learn exercises?”

Probes: features to look for/avoid; how to start safely; how to adapt movements; when to seek guidance.

23. “Which platform tools helped you learn (chapters, captions, playback speed, playlists), and how should they be used in future videos?”

24. “What kind of progression (if any) felt natural or realistic for you over time?”

Probes: session length; frequency; signs you’d use to adjust; when you’d pause.

## I. Integration with Care & Implementation

25. “How would you like clinicians to introduce or brief you before using videos?”

26. “What would make it easier to fit videos into your routine?”

27. “Are there barriers that might stop people from benefiting (costs, login, language, disability access)? What should services change?”

## J. Cultural Fit & Representation (optional)

28. “Did the language, tone, body ideals, or examples feel culturally appropriate to you? What would feel more familiar or respectful?”

## K. Critical Incident

29. “Tell me about a specific moment that really captures your experience — maybe the best or the most frustrating. What made it stand out?”

# 8) Closing Script

“Is there anything important we haven’t talked about that you’d like to add? Thank you for your time and insights. If you’d like to review a brief summary of your interview for accuracy, let us know and we can arrange that.”

# 9) Demographic & Context Checklist (completed off-record)

• Age; sex; education; employment.

• Height/weight (optional, self-reported) for BMI.

• Group-specific: CLBP duration and typical severity (for CLBP group).

• Frequency of video use; platforms used; typical device.

• Self-rated digital literacy (1–5).

• Recent exercise participation (Y/N).

# 10) Interviewer Field-Notes Template (post-session)

• Context: date/time, in-person vs. remote, setting, technical issues.

• Salient observations: affect, hesitations, notable quotes.

• Reflexive memo: interviewer assumptions, surprises, emerging code/theme ideas; potential influence of the interviewer.

• Follow-ups needed: clarifications; member-check availability (if offered); notes for sampling/theoretical sufficiency.

# 11) Accessibility and Adaptation Notes

• Offer captions/subtitles during remote interviews if helpful.

• Provide plain-language explanations for technical terms (e.g., “tempo,” “modification”).

• Encourage participants to describe adaptations they would make (no demonstration required).

# 12) Probing and Flow Guidance (for Interviewers)

Start broad → move to specifics; avoid leading language.

Neutral prompts: “Could you say more?” “What makes you think that?” “Can you give a recent example?”

Allow silence for reflection. If a question is already covered spontaneously, acknowledge and proceed. Prioritize depth over breadth; not all probes are required.
