# Supplementary material for: A qualitative exploration of video-based motor action observation perceptions in patients with chronic low back pain and asymptomatic participants: An interpretative phenomenological analysis
Source: PLoS One. 2026 Feb 6;21(2):e0326638. doi: 10.1371/journal.pone.0326638 (PMC12880644; doi:10.1371/journal.pone.0326638)
Supplement: S2 File — This audit trail summarizes analytic decisions, consensus procedures, and reflexive memos across the seven-phase IPA workflow, aligned with COREQ/SRQR. It complements the Methods and provides a transparent account of how codes evolved into sub-themes and themes. (DOCX) [file pone.0326638.s002.docx]

**S1 File. Audit Trail Summary**

**1) Corpus, Roles, and Versioning**

- Corpus: 20 semi-structured interviews (10 CLBP; 10 asymptomatic); 29–55 min; verbatim transcription.
- Roles: Two primary coders (independent first cycle), one moderator for consensus; PI oversaw audit log.
- Versioning: Codebook v0 (pilot sensitization) → v1 (first five interviews) → v2 (all cases) → v2.1 (final polish).

**2) Seven-Phase Analytic Flow (Evidence Hooks)**

Phase 1 (Immersion) → Phase 2 (Open coding) → Phase 3 (Experiential statements) → Phase 4 (Patterning) → Phase 5 (Theme mapping) → Phase 6 (Case-by-case repetition) → Phase 7 (Group-level synthesis). Evidence hooks: exemplar quotes flagged in ATLAS.ti; memos tagged to nodes.

**3) Codebook Evolution and Boundary Rules**

- Distinction rule: ‘Evaluation of instruction’ (delivery quality: goals/safety cues, pacing, viewpoint, modeling) vs. ‘Action comprehension’ (viewer’s internal simulation/understanding of mechanics). In boundary cases, we coded the primary locus and created a cross-reference note.
- Subtheme refinements: split ‘Motivation’ to capture quick wins vs. credibility signals; merged overlapping ‘fear of bending’ and ‘fear of load’ under ‘fear of movement’ with tempo noted as amplifier.
- Context moderators: consolidated digital literacy, platform conventions, and parasocial/credibility cues.

**4) Consensus and Discrepancy Resolution (Concrete Examples)**

Example A (Boundary case—delivery vs. comprehension): Quote “I wish the videos were slower or had versions for beginner users.” Coder 1 → ‘Action comprehension’; Coder 2 → ‘Evaluation of instruction’. Resolution: primary concern is delivery (pace/options), which secondarily affects comprehension → coded under ‘Evaluation of instruction’ with a memo linking to ‘Action comprehension’.
Example B (Fear vs. Perceived demand): Quote “Some moves seem beyond me; I’d need an easier version.” Coder 1 → ‘Fear of movement’; Coder 2 → ‘Perceived demand’. Resolution: coded under ‘Perceived demand’ because the statement anticipates difficulty rather than explicit threat; added note: ‘when demand framed as risk, check Fear node’.

**5) Trustworthiness Procedures (Focused Set)**

- Credibility: independent dual coding; scheduled consensus meetings; exemplar-quote checks against raw audio.
- Dependability: dated changelog for code definitions; theme clustering rationales; storage of intermediate maps.
- Confirmability: reflexive memos on assumptions (e.g., expectations about exercise safety) and how they were bracketed.
- Transferability: maximum-variation sampling; thick description of setting and viewing context in manuscript.

**6) Reflexive Highlights (Illustrative Memos)**

Memo 13: We tended to overweight biomechanical clarity; after peer debrief, we foregrounded how explicit safety messaging was a precondition for reappraisal from threat → opportunity.
Memo 21: We initially collapsed viewpoint into pacing; separated after noticing egocentric angles reduced perceived demand.

**7) Saturation / Information Power**

No novel subthemes appeared in the final interviews; stability checks on the last three cases confirmed redundancy. Information power judged sufficient given study aim specificity, sample specificity, and high-quality interview material.

**8) Deviations from Plan and Rationale**

- Added probes post-pilot on explicit safety cues, graded options, pacing/tempo, and point-of-view to better capture delivery features; pilot data excluded from analysis.
- Adopted indicative counts (CLBP/AP) to convey prevalence while maintaining qualitative emphasis.

**9) Linkage to Results and Practice Guidance**

Theme chain formalized as: Delivery/Representation → Action Comprehension → Threat/Opportunity Appraisal → Uptake/Adherence, moderated by Pain history, Self-efficacy, Familiarity, and Digital/Platform literacy. This structure underpins practice tactics (safety-first scaffolding; instructional minimalism; contextual tailoring/credibility).
