# Supplementary material for: A qualitative exploration of video-based motor action observation perceptions in patients with chronic low back pain and asymptomatic participants: An interpretative phenomenological analysis
Source: PLoS One. 2026 Feb 6;21(2):e0326638. doi: 10.1371/journal.pone.0326638 (PMC12880644; doi:10.1371/journal.pone.0326638)
Supplement: S1 Table — This table summarizes themes, sub-themes, analytic definitions, exemplar quotations, indicative counts by group, and the explicit linkage to delivery/representation features identified in the manuscript (“Instructional delivery as the gateway to understanding”). (DOCX) [file pone.0326638.s003.docx]

**S1 Table. Codebook and Thematic Summary**

| Theme | Sub-theme | Analytic definition | Exemplar quotation (ID) | Indicative counts (CLBP / AP) | Delivery/Representation link | Notes |
| --- | --- | --- | --- | --- | --- | --- |
| Emotional & Motivational Impact | Fear of movement | Anticipatory threat appraisals (flexion, load, rapid tempo) shaping willingness to try. | PP10: “Watching some exercises, I worry I’ll do them wrong and flare my pain.” | 6/10 \| 1/10 | Threat ↑ when pace fast; flexion/loading cues; unclear safety messages. | Clustered around perceived spinal flexion, axial loading, rapid tempo. |
|  | Avoidance beliefs/behaviors | Protective rules and avoidance scripts triggered by perceived risk. | PP7: “If a movement looks risky, I’d rather skip it.” | 8/10 \| 3/10 | Avoidance ↑ when no graded options; no beginner variant shown. | Risk framing and lack of modifications sustained avoidance. |
|  | Motivation for movement | Encouragement contingent on perceived safety, relevance, and adaptability. | AP10: “Seeing clear demonstrations makes me want to try them.” | 9/10 \| 10/10 | Motivation ↑ with clear goals, explicit safety cues, credible modeling. | Quick wins and relevance cues supported motivation. |
| Self-Assessment of Physical Capacity | Competence | Confidence to execute safely/effectively, often when graded options/safety cues present. | PP1: “I can adapt the exercise… that gives me confidence.” | 4/10 \| 5/10 | Competence ↑ with level-tagging, beginner-first progressions. | Adaptability cues (modifications) were pivotal. |
|  | Perceived demand | Anticipated difficulty/workload based on pace, point-of-view, modeling fidelity. | PP4: “Some moves seem beyond me; I’d need an easier version.” | 7/10 \| 6/10 | Demand ↓ with slower pacing and accurate modeling. | Fast tempo/camera cuts inflated perceived demand. |
| Cognitive Movement Assessment | Attention to activity | Attention to posture, tempo, breathing, error-avoidance cues during viewing. | PP9: “I watch posture and tempo closely to understand the movement.” | 6/10 \| 7/10 | Salient cues ↑ comprehension when on-screen and verbal cues aligned. | Micro-cues (breathing/tempo) were frequently cited. |
|  | Action comprehension | Understanding mechanics/sequence; ability to run an internal simulation. | PP6: “Without step-by-step explanation, I don’t fully understand…” | 5/10 \| 8/10 | Comprehension ↑ with goal clarity, stepwise structure, annotated errors. | Ambiguity in instruction → rewatching and hesitation. |
|  | Evaluation of instruction | Judgments about delivery (clarity of goals/safety cues, pacing, viewpoint, modeling). | PP3: “Beginner versions and slower pace would help me follow along.” | 4/10 \| 6/10 | Positive evaluations with explicit safety/‘why it’s safe,’ chapters, speed control. | Boundary rule distinguished from ‘Action comprehension’ in analysis. |
|  | Familiarity with the motor action | Prior exposure facilitating recognition/simulation; unfamiliar tasks prompt caution. | AP9: “Because I’ve done something similar, it’s easier to picture and try.” | 4/10 \| 5/10 | Familiarity × clarity synergy → smoother simulation. | Unfamiliar sequences → replay and verification. |
