# Supplementary material for: A qualitative exploration of video-based motor action observation perceptions in patients with chronic low back pain and asymptomatic participants: An interpretative phenomenological analysis
Source: PLoS One. 2026 Feb 6;21(2):e0326638. doi: 10.1371/journal.pone.0326638 (PMC12880644; doi:10.1371/journal.pone.0326638)
Supplement: Attachment — Submitted filename: Response two reviwers_Plos_One.docx [file pone.0326638.s006.docx]

**Response to Reviewers**

We thank the Academic Editor and Reviewer #1 for their constructive feedback on our manuscript (PONE-D-25-26151), *“A Qualitative Exploration of Video-Based Motor Action Observation Perceptions in Patients with Chronic Low Back Pain and Asymptomatic Participants: An Interpretative Phenomenological Analysis.”*

We have revised the manuscript accordingly. Below we respond point-by-point; section and line numbers correspond to the tracked-changes file in the revision.

**ACADEMIC EDITOR (FROM EMAIL)**

Weaknesses & Areas for Improvement

1.Abstract - quite detailed - could be written more briefly for better readability Too much attention to background vs. results.

**Response.** Addressed. We condensed the background to two concise sentences and rebalanced the abstract to emphasize results and implications.

**Revised Abstract**

Background. Video-based action observation (AO) of exercise/motor-action content is increasingly delivered via social media. This expands reach and ecological validity but may shape motor simulation, perceived safety, and engagement. How people with chronic low back pain (CLBP) interpret and intend to use such videos remains underexplored.

Methods. We conducted an interpretative phenomenological analysis (IPA) of semi-structured interviews with purposively sampled adults (n=20; 10 CLBP, 10 asymptomatic). Interviews probed perceptions of exercise/motor-action AO videos drawn from common platforms. Analysis followed IPA procedures with iterative coding, constant comparison, and team reflexivity, and is reported according to COREQ.

Results: Three interrelated themes were identified: (1) Emotional & motivational impact: CLBP participants frequently appraised bending, load and fast tempo as threatening and described protective avoidance rules. Motivation was present in both groups when videos felt safe and adaptable; (2) Self-assessment of physical capacity: Perceived competence increased when videos provided graded options and explicit safety cues. Anticipated task demand decreased with appropriate pacing/tempo, egocentric viewpoint, and credible modeling; (3) Cognitive movement assessment: Viewers attended to posture, tempo, breathing and error-avoidance cues. Action comprehension faltered when instructions were dense/fast or goals were. Judgments about delivery (goal clarity, safety cues, pacing, viewpoint, modeling fidelity) shaped internal rehearsal and willingness to attempt.

Conclusions. Individuals with and without CLBP perceive social-media–delivered exercise AO as useful when videos are tailored (graded options, clear safety messaging, appropriate pacing/viewpoint) and contextualized to pain-related concerns and digital literacy. These insights inform clinically oriented AO exercise-video libraries and implementation strategies (e.g., curated playlists, level-tagging, therapist-mediated briefing) to enhance acceptability and adherence in CLBP rehabilitation.

2.Introduction - Although comprehensive, it's long and dense. Key concepts (fear of movement, motor simulation, social media delivery) could be stream-lined for simplicity.

**Response:** Addressed. We condensed the neuroscience/mirror-neuron sections into a single paragraph, removed repeated social-learning content, and added an explicit gap statement linking social-media delivery of exercise/motor-action AO to patients’ perceptions and adherence, followed by a concise rationale for IPA.

We also integrated the constructs into one conceptual paragraph that frames how AO videos may modify threat appraisal and motor simulation in CLBP, removing redundancies.

3.Methods

o Sampling strategy is purposive (but rationale for final sample size (that is, n=20) could be elaborated upon beyond citation.

Response. We justify n = 20 using information power and IPA’s idiographic depth, and describe procedural saturation (no emergent subthemes toward the end of data collection), documented in the audit trail.
Change in manuscript. Methods: Sampling and Participants / Data Analysis (justification added).

o Mentioned is pilgrimage "pilot testing of interview guide but do not include of influence of pilot test interview guide to the final version"

Response. We describe modifications after the pilot (simplified wording; added probes on safety cues, pacing/tempo, and viewpoint) and note that pilot data were not included in analysis.
Change in manuscript. Methods: Interview Guide and Pilot Testing (expanded).

o Reflexivity is mentioned but the positionality of researchers are not explicitly discussed

Response. Addressed. We added a dedicated Positionality and Reflexivity subsection describing each researcher’s disciplinary background and prior clinical/research experience with CLBP and AO, anticipated assumptions, and bias-mitigation strategies (reflexive journaling, peer debriefing, iterative memoing, negotiated consensus).
Change in manuscript. Methods: Positionality and Reflexivity (new subsection).

4.Results

o	Very clear tables that are slightly overloaded; consider simplifying, or moving detailed demographics from tables to supplementary material.

Response: Addressed. Table 2 now presents only core variables, and duplicative narrative around those variables was removed. Quotation density was reduced; we retained non-overlapping, illustrative excerpts per sub-theme.
We introduced Table 2 to compactly summarize themes, sub-themes, definitions, exemplar quotations, and indicative counts, and we reduced quotations to essential, non-overlapping examples.

- Quotes are descriptive, but there is some redundancy-some of the quotes are less important-it may work better to have fewer, more impactful quotes.

Response: We consider it is worthwhile to maintain the texts in order to provide the work with the greatest possible clarity and explanation.

5.Discussion

o Tends to reiterate the results and not carry out deep interpretations based on connections with broader literature.

Response. We rewrote the opening to present an interpretive synthesis rather than a recap. The section now foregrounds three cross-cutting threads—threat vs opportunity appraisal, instructional delivery as gateway to understanding, and contextual moderators—to explain how and why participants read AO videos as signals of safety, competence, and credibility.
Change in manuscript. Discussion, opening two paragraphs.

o James Herriot: "Can benefit from better linkage to clinical application - how should clinicians really apply these findings?"

Response: We added a Practice guidance subsection with three tactics: (1) safety-first scaffolding, (2) instructional minimalism with explicit goals and one error cue, and (3) contextual tailoring/credibility (clinic co-branding, captions, chapters, speed control). Each tactic is linked to the interpretive mechanism (reframing threat → opportunity).
Change in manuscript: Discussion, Practice guidance derived from the IPA, items 1–3.

o Limitations are recognized and not discussed about the potential impact of cultural context (Spanish participants, digital literacy differences)

Response: We added a Cultural transferability and equity subsection addressing our single-country context, variability in platform literacy, and cultural differences in tone and instructional style. We link these issues to recurring implementation determinants for MSK digital services—governance/guidelines, workflow integration, stakeholder acceptance/usability, and financing/incentives—relevant to deploying AO videos in routine practice (van Tilburg et al., 2024).
Change in manuscript: Discussion, Cultural transferability and equity considerations.

6.Conclusion - Good summary but could try to underline contribution to future work for intervention development more strongly

Response: We rewrote the Conclusion to (i) synthesize the interpretive contribution in one compact paragraph (what AO videos *mean* to people with and without CLBP), and (ii) add a forward-looking paragraph translating those insights into an actionable template for AO-based intervention development (safety-first scaffolding, instructional minimalism, contextual tailoring/credibility). We explicitly link these recommendations to plausible adherence mechanisms (threat → opportunity reappraisal; comprehension gains) and to implementation needs highlighted in recent digital MSK literature.
Change in manuscript: Conclusion, entire section replaced (two concise paragraphs).

7.Language & Style - Usually clear but sometimes very wordy; tighten some of the sentences for conciseness.

Response: The new Conclusion avoids restating sub-themes and instead states the core interpretive take-home messages and their clinical/implementation implications in <200 words.

8.Referencing - Comprehensive, but some old references; inclusion of latest (2022-2024) study on digital rehabilitation can hugely improve the relevance.

Response: We integrated recent evidence that structured, credible remote guidance improves outcomes in back pain programs (Geraghty et al., 2024; Cui et al., 2023). We connected our findings to platform dynamics influencing intentions via creator credibility and modeling (Durau et al., 2022; Kim et al., 2024) and to parasocial relationships that bolster self-efficacy when clinicians curate/co-brand content (Chauvin et al., 2024). We aligned implementation implications with a system-level review of MSK digital services (van Tilburg et al., 2024).
Change in manuscript: Discussion, paragraphs “Threat appraisal…”, “Instructional delivery…”, “Contextual moderators…”, “Practice guidance…”, and “Cultural transferability…”.

**Suggestions for Improvement**

Shortening and sharpening Abstract and Introduction.

Response: We have done it.

(or) "How did you manage researcher reflexivity?" "What biases did you have?"

Response. Addressed. We added a dedicated Positionality and Reflexivity subsection describing each researcher’s disciplinary background and prior clinical/research experience with CLBP and AO, anticipated assumptions, and bias-mitigation strategies (reflexive journaling, peer debriefing, iterative memoing, negotiated consensus).
Change in manuscript. Methods: Positionality and Reflexivity (new subsection).

Examples of assertions include the following: Documents Add some detail on trustworthiness strategies (credibility, dependability, confirmability, transferability).

Response: Addressed. We now detail a focused set of procedures: dual independent coding with consensus meetings (credibility), an analytic decision log/audit trail (dependability), reflexive memos and retention of the evolving codebook/theme maps (confirmability), and maximum-variation purposive sampling with contextual description (transferability).
Change in manuscript: Methods: Trustworthiness (new subsection).

Balance Writing Presentation: -Streamline You must present your results in a narrative way and also illustrate with quotes to explain what's happening.

Response: Addressed. Results paragraphs were tightened; Table 2 mirrors the textual structure (theme → sub-theme → definition → quote → counts) for easy cross-reference.

**REVIWER (FROM EMAIL)**

**Review Comments to the Author**

Reviewer #1: This is a well-designed qualitative study exploring how patients with chronic low back pain (CLBP) and asymptomatic individuals perceive video-based motor action observation (AO). The study addresses an important research gap and follows rigorous qualitative research guidelines (COREQ, SRQR).
However, I suggest a few minor revisions to improve clarity and presentation:

1. Simplify certain sections of the introduction and discussion to avoid verbosity.

Response: We have done it, we have reworded the introduction and discussion.

2. In the Methods, you mentioned using "purposive sampling," but it is not clear whether you considered diversity in factors like gender, age, or digital literacy. Add a sentence explaining how participants were selected to ensure a variety of perspectives.

Response. Implemented. We specify that sampling targeted variation across gender, age strata, CLBP duration/severity, and digital literacy/use of social-media exercise content, and we report the achieved heterogeneity.
Change in manuscript. Methods: Sampling and Participants (expanded).

3. In the Results, reduce repetitive quotes and consider adding a visual thematic map or summary table.
Response: Addressed. We introduced Table 2 to compactly summarize themes, sub-themes, definitions, exemplar quotations, and indicative counts, and we reduced quotations to essential, non-overlapping examples.
4. The discussion could better emphasize practical clinical applications and broader implications of the findings.
Response. We added a Practice guidance subsection with three tactics: (1) safety-first scaffolding, (2) instructional minimalism with explicit goals and one error cue, and (3) contextual tailoring/credibility (clinic co-branding, captions, chapters, speed control). Each tactic is linked to the interpretive mechanism (reframing threat → opportunity).
Where. Discussion, Practice guidance derived from the IPA, items 1–3.
5. Minor grammatical corrections and sentence restructuring are needed for better readability.
Response. We tightened prose throughout the manuscript to maintain an interpretive register consistent with interpretative phenomenological analysis and better clarity.

**REVIWER (FROM WORD FILE)**

**Comments for the Author**

**Abstract**

1. In the abstract, briefly explain why using social media videos is important for this type of study.

**Response.** Addressed. The background now succinctly states why social-media delivery matters and the abstract foregrounds findings and implications.
**Change.** Abstract, Background (first paragraph).

2. Instead of "Study Design," you could label the section as "Methods" to match common structure.

**Response.** Implemented.
**Change.** Abstract subheading changed to **Methods**.

**Introduction:**

1. Try to make introduction section a bit shorter. The parts about brain function and mirror neurons can be summarized briefly.

**Response.** Implemented. Neurophysiology is now one concise synthesis focusing on motor simulation/resonance, with a brief note that mirror neurons are not the sole mechanism.
**Change in manuscript.** Introduction: previous multi-paragraph section replaced by a single compact paragraph.

2. A better link between the background and why this qualitative study is needed would improve the flow.

**Response.** Implemented. We now state that prior AO/exercise-video studies seldom examined *how* people perceive, interpret, and evaluate observed movements and how this shapes engagement; the closing paragraph articulates the gap and the need for IPA.

**Change in manuscript.** Introduction: new bridging sentences and revised final paragraph (gap → aim → qualitative rationale).

**Methods:**

1. You mentioned using "purposive sampling," but it is not clear whether you considered diversity in factors like gender, age, or digital literacy. Add a sentence explaining how participants were selected to ensure a variety of perspectives.

Response. Implemented. We specify that sampling targeted variation across gender, age strata, CLBP duration/severity, and digital literacy/use of social-media exercise content, and we report the achieved heterogeneity.
Change in manuscript. Methods: Sampling and Participants (expanded).

2. You have explained the seven-phase IPA process well, but more clarity is needed on how the codes were developed. Were they entirely **data-driven (inductive) or informed by previous literature (deductive)?**

Response. We state that coding was primarily inductive within an IPA framework, with limited deductive sensitization (e.g., fear of movement, motor simulation, instructional features) used only to orient early memoing; final themes were data-driven.

Change in manuscript. Methods: Data Analysis (clarified).

3. You have mentioned using a semi-structured interview guide, but the guide itself is not provided. Consider adding the **interview guide as a supplementary file**, or state that it is available on request.

Response. Addressed. We describe modifications after the pilot (simplified wording; added probes on safety cues, pacing/tempo, and viewpoint) and note that pilot data were not included in analysis.
Change in manuscript. Methods: Interview Guide and Pilot Testing (expanded).

**Results:**

1. Add a **flowchart** or a **summary table** that shows the three main themes and their sub-themes together for easy reference.

Response: Addressed. We introduced Table 2 to compactly summarize themes, sub-themes, definitions, exemplar quotations, and indicative counts, and we reduced quotations to essential, non-overlapping examples.

2. Some sub-themes (e.g., "Evaluation of Instruction" and "Action Comprehension") seem to overlap in content.

Response: Addressed. We added an explicit operational distinction in Results: Evaluation of instruction refers to delivery features (clarity of goals, explicit safety cues, pacing/tempo, viewpoint, modeling fidelity), while Action comprehension refers to the viewer’s understanding and internal simulation of mechanics/sequence. Boundary cases were coded by primary locus with linkage noted.

Provide a short explanation of how these sub-themes are different.

Response: We now report indicative counts per sub-theme (e.g., 6/10 CLBP; 1/10 asymptomatic for fear related to flexion/load), maintaining a qualitative emphasis.

3. **Table 1 is** very detailed but includes some repetitive information (e.g., gender is mentioned in both text and table). Consider simplifying the text description and refer readers to the table for details, to avoid redundancy.

Response: We reviewed the text to confirm there is not redundancy left.

4. Though this is a qualitative study, some counts (e.g., “X out of 10 CLBP participants expressed fear of bending”) can help understand how common certain themes were. Add numbers or frequencies to show how many participants expressed key concerns (e.g., fear of movement, need for modified exercises).

Response: Addressed. We now report indicative counts per sub-theme (e.g., 6/10 CLBP; 1/10 asymptomatic for fear related to flexion/load), maintaining a qualitative emphasis.

5. The sub-themes within each theme are not consistently ordered by importance or frequency. Consider presenting sub-themes in order of how frequently they appeared in the data (e.g., if “Fear of Movement” was mentioned most, list it first).

Response: Addressed. Sub-themes are presented in order of observed prevalence/centrality, and indicative counts are provided to aid interpretation.

**Discussion:**

1. Discussion section is a bit wordy and repeats some information already mentioned in the Results. Focus more on interpreting the results, not repeating them.

Response. We rewrote the opening to present an interpretive synthesis rather than a recap. The section now foregrounds three cross-cutting threads—threat vs opportunity appraisal, instructional delivery as gateway to understanding, and contextual moderators—to explain how and why participants read AO videos as signals of safety, competence, and credibility.
Change in manuscript. Discussion, opening two paragraphs.

2. While the potential of AO for rehabilitation is mentioned, practical applications (e.g., how physiotherapists can adapt exercise videos for CLBP patients) are not fully discussed. Add 2-3 practical examples of how this research can guide clinical physiotherapy practice.

Response. We added a Practice guidance subsection with three tactics: (1) safety-first scaffolding, (2) instructional minimalism with explicit goals and one error cue, and (3) contextual tailoring/credibility (clinic co-branding, captions, chapters, speed control). Each tactic is linked to the interpretive mechanism (reframing threat → opportunity).
Change in manuscript: Discussion, Practice guidance derived from the IPA, items 1–3.

3. Limitations are discussed, but mention **technology familiarity bias** (some participants may be more comfortable with digital platforms than others).

Response. Strengths and limitations now (i) note that interviews capture intentions/experiences not behavior, (ii) acknowledge the absence of concurrent motor observation, (iii) recognize single-country generalizability limits, (iv) detail technology-familiarity influences on using pause/replay/speed—mirroring implementation challenges reported for MSK apps/programs (Ulrich et al., 2023), and (v) describe reflexive procedures to mitigate interpretive bias.
Change in manuscript: Discussion, Strengths and limitations.
