# Supplementary material for: A qualitative exploration of video-based motor action observation perceptions in patients with chronic low back pain and asymptomatic participants: An interpretative phenomenological analysis
Source: PLoS One. 2026 Feb 6;21(2):e0326638. doi: 10.1371/journal.pone.0326638 (PMC12880644; doi:10.1371/journal.pone.0326638)
Supplement: Attachment — Submitted filename: Reviewer comment.docx [file pone.0326638.s005.docx]

**A Qualitative Exploration of Video-Based Motor Action Observation Perceptions in Patients with Chronic Low Back Pain and Asymptomatic Participants: An Interpretative Phenomenological Analysis**

**Comments for the Author**

**Abstract**

1. In the abstract, briefly explain why using social media videos is important for this type of study.
2. Instead of "Study Design," you could label the section as "Methods" to match common structure.

**Introduction:**

1. Try to make introduction section a bit shorter. The parts about brain function and mirror neurons can be summarized briefly.
2. A better link between the background and why this qualitative study is needed would improve the flow.

**Methods:**

1. You mentioned using "purposive sampling," but it is not clear whether you considered diversity in factors like gender, age, or digital literacy. Add a sentence explaining how participants were selected to ensure a variety of perspectives.
2. You have explained the seven-phase IPA process well, but more clarity is needed on how the codes were developed. Were they entirely **data-driven (inductive) or informed by previous literature (deductive)?**
3. You have mentioned using a semi-structured interview guide, but the guide itself is not provided. Consider adding the **interview guide as a supplementary file**, or state that it is available on request.

**Results:**

1. Add a **flowchart** or a **summary table** that shows the three main themes and their sub-themes together for easy reference.
2. Some sub-themes (e.g., "Evaluation of Instruction" and "Action Comprehension") seem to overlap in content. Provide a short explanation of how these sub-themes are different.
3. **Table 1 is** very detailed but includes some repetitive information (e.g., gender is mentioned in both text and table). Consider simplifying the text description and refer readers to the table for details, to avoid redundancy.
4. Though this is a qualitative study, some counts (e.g., “X out of 10 CLBP participants expressed fear of bending”) can help understand how common certain themes were. Add numbers or frequencies to show how many participants expressed key concerns (e.g., fear of movement, need for modified exercises).
5. The sub-themes within each theme are not consistently ordered by importance or frequency. Consider presenting sub-themes in order of how frequently they appeared in the data (e.g., if “Fear of Movement” was mentioned most, list it first).

**Discussion:**

1. Discussion section is a bit wordy and repeats some information already mentioned in the Results. Focus more on interpreting the results, not repeating them.
2. While the potential of AO for rehabilitation is mentioned, practical applications (e.g., how physiotherapists can adapt exercise videos for CLBP patients) are not fully discussed. Add 2-3 practical examples of how this research can guide clinical physiotherapy practice.
3. Limitations are discussed, but mention **technology familiarity bias** (some participants may be more comfortable with digital platforms than others).

**Comments to the Editor**

This is a well-written qualitative study exploring an emerging area—motor action observation via social media in the context of chronic pain rehabilitation. The manuscript adheres to COREQ/SRQR guidelines and employs robust methods.

I recommend **minor revision** before acceptance, mostly to streamline text, enhance clarity of themes, and expand on reflexivity. These revisions will improve the paper’s readability and impact without requiring major methodological changes.
